# Problematic smartphone use and sleep disturbance: the roles of metacognitions, desire thinking, and emotion regulation

**DOI:** 10.3389/fpsyt.2023.1137533

**Published:** 2023-08-01

**Authors:** Mehdi Akbari, Mohammad Seydavi, Sonay Sheikhi, Marcantonio M. Spada

**Affiliations:** ^1^Department of Clinical Psychology, Faculty of Psychology and Education, Kharazmi University, Tehran, Iran; ^2^School of Applied Sciences, London South Bank University, London, United Kingdom

**Keywords:** desire thinking, emotion regulation, metacognitions about smartphone use, problematic smartphone use, sleep disturbance

## Abstract

**Background:**

The association between problematic Smartphone use (PSU) and sleep disturbance is evidenced in the literature, but more research is required to investigate the potential factors that may influence the effect of PSU on sleep disturbance. Given the considerable prevalence of PSU (9.3 to 36.7%) and sleep disturbance (55.2%) in Iran, the current study sought to examine an interactional model to test whether metacognitions about Smartphone use, desire thinking (verbal perseveration and imaginal prefiguration), and emotion regulation (expressive suppression and cognitive reappraisal) could have a moderating effect on the above-mentioned association.

**Method:**

This present study is a cross-sectional, observational study that was conducted between June and September 2022 in a convenience sample of Iranians (*n* = 603, Female = 419, Age = 24.61 ± 8).

**Results:**

Despite the significant association between metacognitions about the Smartphone use, PSU, and sleep disturbance, metacognitions failed to predict sleep disturbance above PSU. A slope analysis showed, however, that a high (not low or moderate) levels of imaginal prefiguration strengthen the association between PSU and sleep disturbance, while a high (not low or moderate) level of cognitive reappraisal and expressive suppression dampen the PSU-sleep disturbance association. We also found that verbal perseveration and expressive suppression were unique predictors of sleep disturbance, while imaginal prefiguration and reappraisal only predicted sleep disturbance if they interacted with PSU.

**Conclusion:**

Theoretically, findings suggest that enhancing cognitive reappraisal (by 1 SD) and reducing imaginal prefiguration (by 1 SD), might protect against sleep disturbance by reducing its association with PSU. Limitations and future directions are discussed.

## 1. Introduction

The Smartphone is used widely for many aspects of our daily lives, such as communication, entertainment, and paying bills (1). Despite all benefits of a Smartphone, people have been found to be prone to engaging in ‘problematic Smartphone use (PSU)’. This term refers to the excessive, compulsive, and compensatory use of the Smartphone in improper situations (2), and is one of the types of non-chemical behavioral addiction given the incapacity to regulate smartphone use time which could span from mild PSU to a more extreme addictive behavior (3). PSU is related to main components of addiction: tolerance (i.e., increasing Smartphone use to achieve satisfaction), compulsion (overuse), withdrawal (i.e., negative symptoms which occur after Smartphone use discontinuation), mood modification (Smartphone use induces direct alterations in mood), conflict (intrapersonal and interpersonal difficulties stemming from smartphone use), relapse (after a period of abstinence, the return to PSU), and functional impairment (4, 5). PSU can cause adverse consequences in different aspects of a user’s life, including psychological and physical health, and poor sleep quality (6).

Given that the prevalence of PSU is between 9.3 and 36.7% (7–9), and the prevalence of sleep disturbance is in the region of 55.2% (10) and 35.7% (11) in Iran and the globe, respectively, this appears to be an important area of research in behavioral addiction. A recent meta-analysis (7) found that among 41,871 children and young adults (female = 55%) found that PSU could double the odds of sleep disturbance, i.e., the incidence of sleep disturbance among people with PSU is two and a half times higher than among people without PSU, nonetheless, several factors may be associated with the persistence of sleep disturbance although few studies have assessed in depth this issue. In general, sleep disturbance refers to symptoms of chronic insomnia, hypersomnia, excessive daytime lethargy, circadian rhythm disturbance, difficulty falling asleep and/or staying asleep, which are associated with daytime function impairment (12).

PSU may predispose people to sleep disturbance due to several factors including, using Smartphones in bed, which shortens nighttime sleep duration, the light from Smartphones disrupting the circadian rhythm (13), using a mobile phone at night affecting brain activity, particularly the pineal gland, and altering the brain’s electrical activity and cerebral blood flow, all of which have a negative impact on sleep quality (14), reducing rapid eye movement (REM) sleep, longer sleep latency, slow wave sleep (15), shortened sleep duration (16), and suppressing sleep-promoting hormones like melatonin given the blue light emitted from Smartphones (17, 18). Thus, research suggests that people with sleep disturbance should also be screened for PSU (19). Furthermore, the association between PSU and sleep disturbance, may be affected by factors potentially linked to sleep quality or with PSU such as metacognitions, desire thinking, and emotion regulation, which will be discussed below.

Metacognition is a multifaceted concept that is broadly defined (in the psychopathology arena) as knowledge of mental activities (‘metacognitions’) and strategies for appraising, monitoring, and regulating cognition, which can include worry, rumination, thought suppression and threat monitoring, for example (20). Two levels of sleep-related arousal involve metacognition and cognition processes. Primary arousal is related to thoughts about the inability to sleep and secondary arousal consists of amplifying the negative emotions’ valence and/or creating biases in the attention to sleep-related thoughts (21). Metacognitions (beliefs about cognition and how it should be controlled) play a critical role in the etiology and maintenance of sleep disturbance (22, 23). Cognitive intrusive thoughts about sleep (e.g., “thinking in bed means I will not get to sleep” or “thinking in bed prevents me from getting to sleep”) and how to control these unwanted thoughts before sleep will come in (e.g., “Before I fall asleep, I must try to have a restful mind, maybe by using my Smartphone” or “Before I fall asleep, I must try to switch off my thoughts and my Smartphone may help me with it”) are the main focus of people’s metacognition about sleep (24), particularly in the context of PSU.

Recently, Casale and colleagues (2020), have developed the Metacognitions about Smartphone Use Questionnaire which aimed to assess: (i) positive metacognitions about emotional and cognitive regulation (e.g., “My Smartphone helps me control my negative thoughts”) and about socio-cognitive regulation through Smartphone use (e.g., “Using a Smartphone increases my sociability when I am feeling lonely”); and (ii) negative metacognitions about the uncontrollability (e.g., “My Smartphone use is beyond my control”) and cognitive harm arising from Smartphone use (e.g., My mind will be damaged by the use of a Smartphone). Recently, Casale et al. (25) published a systematic review on the association between metacognitions and technological addictions, such as PSU. However, they suggested that additional research on metacognitions in the context of PSU is necessary to provide a more thorough picture of the role of metacognitions in PSU.

Given the association between metacognitions and sleep disturbance (22, 23), and the association between PSU and sleep disturbance (6), we were curious to examine whether there is an interaction between PSU and metacognitions about Smartphone use in predicting sleep disturbance.

*H1*: Metacognitions about Smartphones will interact with PSU in predicting sleep disturbance.

Testing such interaction would be interesting, given the evidence supporting the role of metacognitions in sleep disturbance was assessed by a generic measure of metacognitions (21), and given the role of PSU in sleep disturbance. Furthermore, exploring such interaction using a tailored measure of metacognitions about Smartphone use may provide a deeper understanding of PSU-related sleep disturbance.

Another concept related to PSU is desire thinking. Desire thinking is a transdiagnostic factor in addictive behaviors (26) and can be conceptualized as voluntary cognitive process that is characterized by the conscious elaboration of memories and images about the positive target-related experience (27). Desire thinking is bi-dimensional in nature and encompasses: (i) imaginal prefiguration, which refers to the allocation of attentional resources toward elaborating (through imagery) positive target-related information and (ii) verbal preservation, which refers to prolonged self-talk regarding worthwhile reasons for engaging in positive target-related experience. Recent research by Marino et al. (28) suggests that Smartphone use and social media use are overlapping concepts, given that Smartphones provide access to social media. Consequently, the literature on the association between desire thinking and social media use may establish the groundwork for the potential association between desire thinking and PSU. In addition, research has verified the association between desire thinking and problematic social media use (29, 30) and identified desire thinking as a distinct predictor of problematic social media use (31), to our knowledge, however, no research has examined the association between desire thinking and sleep disturbance. Desire thinking has been associated with negative affect, impulsivity, and thought suppression in relation to desire thinking and problematic social media use (32). Considering the association of desire thinking with problematic social media use and its overlap with PSU, and also the association between PSU and sleep disturbance, we were curious as to whether there would be an interaction between PSU and desire thinking in predicting sleep disturbance. This might shed light on the underlying factors that explain variance in sleep disturbance by keeping the PSU ‘engine’ active. According to the above-mentioned studies, it is of interest, to explore whether the association between PSU with sleep disturbance is moderated by desire thinking.

*H2*: Desire thinking will interact with PSU in predicting sleep disturbance.

The last construct, emotion regulation, has been found to be another predictor of sleep disturbance (33). Emotion regulation is defined as an attempt to modify and control processes involved in the initiation, duration, and maintenance of negative and positive emotions (34). Some emotion regulation strategies such as problem-solving, acceptance and cognitive appraisal, and distraction, appear to be adaptive in some contexts while other strategies such as suppression, rumination, and avoidance appear to exacerbate emotional distress (35). Research has shown that cognitive reappraisal, defined as the re-evaluation of emotional eliciting stimuli to change their emotional relevance, and expression suppression, characterized by an active attempt to inhibit the behavioral expression of an emotional experience, are related to sleep quality (33). Considering the associations between both emotion regulation strategies and PSU on the one hand and sleep disturbance on the other, it would be interesting to explore whether is there an interaction between PSU and emotion regulation strategies (cognitive reappraisal and expressive suppression) in predicting sleep disturbance. It is of interest, as those higher on reappraisal and lower on expression suppression appear to be more prone to psychological distress, experiencing negative emotions and low quality of life (36). Thus, testing the interaction between PSU and emotion regulation strategies may provide insights into the extent to which different levels of emotion regulation might affect the association between PSU and sleep disturbance.

*H3*: Emotion regulation will interact with PSU in predicting sleep disturbance.

Given that PSU is a predictor of sleep disturbance and that metacognitions about smartphones, desire thinking, and emotion regulation are associated with PSU, it was intriguing to investigate whether these variables interact with PSU in predicting sleep disturbance. Although it is possible to merely use hierarchical regression analysis or a multiple regression model to explore what other variables could predict sleep disturbance above and beyond PSU, such analysis is based on average score and would not tell us how the association between PSU and sleep disturbance would differ according to variations in the variables of interest. Such understanding could be accomplished by developing an interactional model using slope analysis to explore whether the possible differences in the association between PSU and sleep disturbance are moderated by other variables. An interactional model examines “when” or “for whom” a variable strongly influences an outcome variable (37), which may provide us with new theoretical insights (38). Given that the presence of an interaction indicates that the association between each of the interacting variables and a third “dependent variable” depends on the value of the other interacting variable, it may have clinical implications regarding the optimal therapeutic target, which variable should be intervened on at what level, and for whom.

## 2. Method

This present study is a cross-sectional, observational study that was conducted between June and September 2022 in a convenience sample of Iranians.

### 2.1. Measures

#### 2.1.1. Pittsburgh sleep quality index scale (PSQI)

The PSQI (39) is a self-report measure of the quality and pattern of sleep over a 1-month interval. It contains 12 items which are scored on a 4-point Likert scale, from 0 (not in the past month) to 3 (three or more times per week). The PSQI has 7 factors: subjective sleep quality, sleep latency, sleep duration, habitual sleep efficiency, sleep disturbances, use of sleeping medication, and daytime dysfunction. The Persian version of this measure indicated good internal consistency (40). In the current study, the McDonald’s omega (ω) of internal consistency was 0.70. Higher scores on PSQI represent higher sleep disturbance.

#### 2.1.2. Emotion regulation questionnaire (ERQ)

The ERQ (41) is a self-report measure of emotion regulation strategies. It contains 10 items which are scored on a 7-point Likert scale, from 1 (strongly disagree) to 7 (strongly agree). The ERQ has two factors: cognitive reappraisal and expressive suppression. The Persian version of this measure indicated good internal consistency (42). In the current study, the McDonald’s omega (ω) of internal consistency for cognitive reappraisal and expressive suppression were 0.82 and 0.75, respectively. Higher scores on cognitive reappraisal and expressive suppression represent higher cognitive reappraisal and higher expressive suppression, respectively.

#### 2.1.3. Metacognitions about smartphone use questionnaire (MSUQ)

The MSUQ (43) is a self-report measure of positive and negative metacognitions about Smartphone use. It contains 24 items which are scored on a 4-point Likert scale, from 1(do not agree) to 4 (agree very much). The MSUQ has three factors: positive metacognitions about emotional and cognitive regulation (MSUQ – PM ECR) through Smartphone use, positive metacognitions about the socio-cognitive regulation (MSUQ – PM SR) through Smartphone use, and negative metacognitions about the uncontrollability and cognitive harm (MSUQ – NM UH) of Smartphone use. The Persian version of this measure indicated good internal consistency (44). In the current study, the McDonald’s omega (ω) of internal consistency for MSUQ – PM ECR, MSUQ – NM UH, and MSUQ – PM SR were 0.94, 0.93, and 0.77, respectively. Higher scores on MSUQ – PM SR represent higher positive metacognitions about emotional and cognitive regulation. Higher scores on MSUQ – PM SR represent higher positive metacognitions about the socio-cognitive regulation. Higher scores on MSUQ – NM UH represent higher negative metacognitions about uncontrollability and cognitive harm.

#### 2.1.4. Desire thinking questionnaire (DTQ)

The DTQ (45) is a self-report measure of desire thinking. It contains 10 items which are scored on a 4-point Likert scale from 1 (almost never) to 4 (almost always). The DTQ has two factors: verbal perseveration and imaginal prefiguration. The Persian version of this measure indicated good internal consistency [but item 10 from the verbal perseveration factor is removed from the Persian version due to low loading; (46)]. In the current study, the McDonald’s omega (*ω*) of internal consistency for verbal perseveration and imaginal prefiguration were 0.89 and 0.87, respectively. Higher scores on verbal perseveration and imaginal prefiguration represent higher verbal perseveration and imaginal prefiguration.

#### 2.1.5. Smartphone addiction scale-short version (SAS-SV)

The SAS-SV (47) is a self-report measure of Smartphone addiction in adolescents and adults. It contains 10 items which are scored on a 6-point Likert scale, from 1 (completely disagree) to 6 (completely agree). The Persian version of this measure indicated good internal consistency (48). In the current study, the McDonald’s omega (*ω*) of internal consistency was 0.88. Higher scores on SAS-SV represent higher PSU.

### 2.2. Participants, procedure, and data analysis

The sample of this study included 603 participants (males = 184, females = 419) aged from 18 to 55 years with a mean age of 24.61 (SD = 8) years. 67.5% of the sample had educational attainment at university level, 46% were employed, 85% were passive users, and 83% were using Smartphones for more than 3 h per day (Please see Table 1 for more information). The participants were recruited voluntarily with no inceptions for participation, using an anonymous online survey advertised on popular social media in Iran (i.e., WhatsApp, Telegram, Twitter, and Facebook) describing the aim of the study. Inclusion criteria were: (i) being able to read and write in Persian; (ii) being resident in Iran; (iii) and being 18 years of age or older.

**Table 1:** Demographic features of the participants (*N* = 603).

	*N* (%)
Gender	
Male	184 (31%)
Female	419 (69%)
Educational attainment	
Less than Diploma	42 (7.0%)
Diploma	154 (26%)
Associate degree	48 (8.0%)
Bachelor degree	220 (36%)
Master Degree	106 (18%)
Ph.D.	33 (5.5%)
Occupational status	
Unemployed	325 (54%)
Part-time	122 (20%)
Full-time	156 (26%)
Type of smartphone use	
Active	93 (15%)
Passive	510 (85%)
Smartphone use time spent	
1 h\day	7 (1.2%)
2 h\day	97 (16%)
3 h\day	200 (33%)
4 h\day	299 (50%)

We inquired as to whether the participants are active on social media if they post frequently, manage online groups or channels in addition to their regular activities. In addition, we inquired as to whether they are passive on social media if they only engage in routine activities such as viewing others’ posts, clips, conversations, etc.

Once participants had been informed about the study and provided informed consent, they completed the study questionnaires. All participants were assured that their data would be kept confidential. Answering all questions was mandatory, therefore, there was no missing data. The study was conducted in alignment with the Declaration of Helsinki (49) for research with human participation.

Before analyzing data for regression analyses, assumptions were tested. The Mahalanobis distance scores identified no multivariate outliers. The multicollinearity statistics were within acceptable limits for the model. The residual analysis (including Loess line fitting and Q-Q plots), scatterplots, and statistic coefficients demonstrated that the normality, linearity, and homoscedasticity assumptions were met. Data were analyzed using IBM SPSS (version 26) and Jamovi (version 2.3.18).

## 3. Results

As seen in Table 2, all variables are normally distributed as measured by skewness and kurtosis indices. According to a Pearson Product–moment set of correlation analyses, sleep disturbance was significantly and positively associated with PSU, desire thinking, metacognitions about Smartphone use, and emotion regulation (except the cognitive reappraisal factor). Moreover, PSU was significantly and positively associated with desire thinking, metacognitions about Smartphone use, and emotion regulation (except the expressive suppression factor).

**Table 2:** The means, standard deviation, kurtosis, skewness, and method of moment correlations of the variables.

Variable	*M*	SD	Range	Skewness	Kurtosis	1	2	3	4	5	6	7	8	9
Sleep disturbance (1)	10.6	5.7	0–36	0.56	−0.09	–	0.22^**^	0.12^**^	0.18^**^	0.08^*^	0.28^**^	0.22^**^	0.10^**^	−0.06
PSU (2)	35.7	12.1	10–60	−0.13	−0.67		–	0.38^**^	0.77^**^	0.34^**^	0.59^**^	0.68^**^	0.06	−0.08^*^
MSUQ – PM ECR (3)	25.5	9.1	11–44	0.33	−0.83			–	0.30^**^	0.69^**^	0.35**	0.43^**^	0.12^**^	0.09^*^
MSUQ – NM UH (4)	21.7	9.0	10–40	0.48	−0.99				-	0.27^**^	0.57^**^	0.65^**^	0.03	−0.10^**^
MSUQ – PM SR (5)	6.7	2.6	3–12	0.35	−0.80					-	0.32^**^	0.39^**^	0.03	0.08^*^
Verbal Perseveration (6)	6.4	3.1	4–16	1.57	1.84						–	0.84^**^	0.08^*^	−0.04
Imaginal Prefiguration (7)	9.1	3.8	5–20	1.18	0.78							–	0.08	−0.04
Expressive Suppression (8)	16.4	4.0	4–28	−0.10	−0.15								–	0.37^**^
Cognitive Reappraisal (9)	25.9	6.7	6–42	−0.35	−0.05									–

PSU, Problematic Smartphone Use; MSUQ – PM ECR, Positive Metacognitions about Emotional and Cognitive Regulation; MSUQ – NM UH, Negative Metacognitions about Uncontrollability and Cognitive Harm; MSUQ – PM SR, Positive Metacognitions about Socio-cognitive Regulation. ^**^ *p* < 0.01; ^*^ *p* < 0.05.

Table 3 presents the results for predicting sleep disturbance scores from PSU (step 1), metacognitions about Smartphone use factors (step 2), desire thinking factors (step 3), and emotion regulation factors (step 4). The results indicated that PSU significantly predicted sleep disturbance, *F* (1, 601) = 31.65, *p* < 0.01, explaining a 5% variance. The addition of metacognitions about Smartphone use factors (step 2) resulted in a significant regression equation, *F* (4, 598) = 8.297, *p* < 0.001, explaining an extra 0.03% of the variation in sleep disturbance scores, ∆*F* (3, 598) = 0.535, *p* = 0.659, which was non-significant (Cohen’s *f* = 0.003). The inclusion of desire thinking factors (step 3) produced a significant equation, *F* (6, 596) = 10.242, *p* < 0.001, accounting for an additional 4.1% of the variation explained in sleep disturbance scores, ∆*F* (2, 596) = 13.441, *p* < 0.001 (Cohen’s *f* = 0.044). Finally, the addition of the emotion regulation factor (step 4) resulted in a significant equation, *F* (8, 594) = 8.936, p < 0.001, accounting for an additional 4.1% of the variation in sleep disturbance scores, ∆*F* (2, 594) = 4.642, p < 0.001 (Cohen’s *f* = 0.015). The final model revealed that predicted variability in sleep disturbance scores was 14.0%, and it was small in term of Cohen’s *f* (< 0.20). In this model, the verbal perseveration factor of desire thinking predicted sleep disturbance scores above and beyond PSU, metacognitions about Smartphone use, and emotion regulation. Additionally, it is imperative to remark that PSU was no longer a significant predictor of sleep disturbance after adding desire thinking and emotion regulation.

**Table 3:** Hierarchical regression model for predicting sleep disturbance.

Predictor	*B 95%CI*[Ul, Ll]	*β*	*T*	*Sr^2^*	*R*	*R*^2^	Adjusted *R*^2^	∆*R*^2^
*Step 1*					0.224	0.050	0.048	0.050^*^
PSU	0.10 [0.06, 0.14]	0.22^*^	5.63	0.22				
			
*Step 2*					0.229	0.053	0.046	0.003
PSU	0.08 [0.02, 0.14]	0.19^*^	2.88	0.11				
MSUQ – PM ECR	0.04 [−0.02, 0.11]	0.06	1.14	0.04				
MSUQ – NM UH	0.01 [−0.05, 0.09]	0.03	0.48	0.01				
MSUQ – PM SR	−0.06 [−0.30, 0.17]	−0.03	−0.57	−0.02				
*Step 3*					0.306	0.093	0.084	0.041^*^
PSU	0.06 [0.003, 0.12]	0.14^*^	2.07	0.08				
MSUQ – PM ECR	0.03 [−0.03, 0.10]	0.05	0.91	0.03				
MSUQ – NM UH	−0.01 [−0.09, 0.06]	−0.03	−0.42	−0.01				
MSUQ – PM SR	−0.09 [−0.32, 0.14]	−0.04	−0.76	−0.03				
Verbal Perseveration	0.61 [0.35, 0.88]	0.34^*^	4.63	0.18				
Imaginal Prefiguration	−0.12 [−0.45, 0.03]	−0.14	−1.71	−0.07				
*Step 4*					0.328	0.107	0.095	0.014^*^
PSU	0.06 [−0.002, 0.12]	0.13	1.92	0.07				
MSUQ – PM ECR	0.02 [−0.04, 0.09]	0.04	0.72	0.02				
MSUQ – NM UH	−0.01, [−0.09, 0.06]	−0.03	−0.48	−0.02				
MSUQ – PM SR	−0.05 [−0.28, 0.18]	−0.02	−0.42	−0.01				
Verbal perseveration	0.60 [0.34, 0.86]	0.33^*^	4.56	0.18				
Imaginal Prefiguration	−0.12 [−0.45, 0.02]	−0.14	−1.73	−0.07				
Suppression	0.16 [0.04, 0.28]	0.12^*^	2.73	0.11				
Reappraisal	-0.08 [-0.15, -0.01]	−10^*^	−2.29	−09				

PSU, Problematic Smartphone Use; MSUQ – PM ECR, Positive Metacognitions about Emotional and Cognitive Regulation; MSUQ – NM UH, Negative Metacognitions about Uncontrollability and Cognitive Harm; MSUQ – PM SR, Positive Metacognitions about Socio-cognitive Regulation. * *p* < 0.05.

### 3.1. Moderator analysis

In addition to the above analysis, we examined whether the significant predictors (desire thinking and emotion regulation) of sleep disturbance would be predicted by their different levels, i.e., average (mean), one standard deviation above the mean, and one standard deviation below the mean. However, given the non-significant equation for metacognitions about Smartphone use predicting sleep disturbance scores, the moderating effect for this variable was not investigated.

#### 3.1.1. Expressive suppression as a moderator

**Figure 1:**
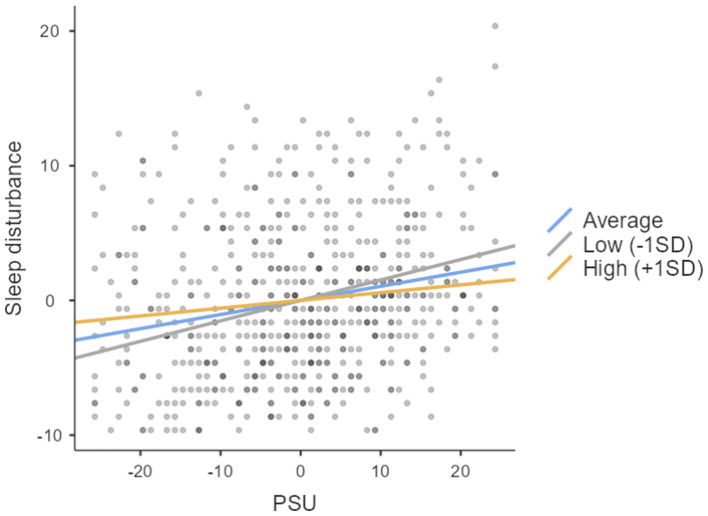
The moderating role of expressive suppression at different levels.

As seen in Table 4, expressive suppression positively and significantly predicted sleep disturbance. Also, it significantly moderated the association between PSU and sleep disturbance. The slope at the low level of expressive suppression was (0.15), at a high level, it was (0.06), and at the average level, it was (0.10). Figure 1 depicts the moderating role of expressive suppression at different levels. This suggests expressive suppression’s strongest influence is at its low and average level.

**Table 4:** The moderation analysis, sleep disturbance as the dependent variable.

Variable	*B 95%CI* [Ul, Ll]	SE	*Z*	*p*
Model 1 – Suppression as a moderator				
PSU	0.10 [0.06, 0.14]	0.0202	5.15	< 0.001
Suppression	0.11 [0.01, 0.22]	0.0556	2.09	0.037
PSU * Suppression	−0.01 [−0.02, −0.002]	0.0049	−2.29	0.022
Model 2 – Reappraisal as a moderator				
PSU	0.10 [0.06, 0.14]	0.0204	5.14	< 0.001
Reappraisal	−0.04 [−0.10, 0.02]	0.0344	−1.28	0.200
PSU * Reappraisal	−0.007 [−0.01, −0.001]	0.0028	−2.50	0.013
Model 3 – Verbal Perseveration as a moderator				
PSU	0.043 [0.006, 0.07]	0.0243	1.77	0.076
Verbal perseveration	0.39 [0.25, 0.54]	0.1173	3.40	< 0.001
PSU * Verbal perseveration	0.003 [−0.006, 0.01]	0.0079	0.41	0.677
Model 4 – Imaginal Prefiguration as a moderator				
PSU	0.082 [0.04, 0.11]	0.0262	3.15	0.002
Imaginal prefiguration	0.068 [−0.04, 0.18]	0.0983	0.69	0.486
PSU * Imaginal prefiguration	0.012 [0.003, 0.02]	0.0059	2.06	0.039

PSU, Problematic Smartphone Use.

#### 3.1.2. Cognitive reappraisal as a moderator

**Figure 2:**
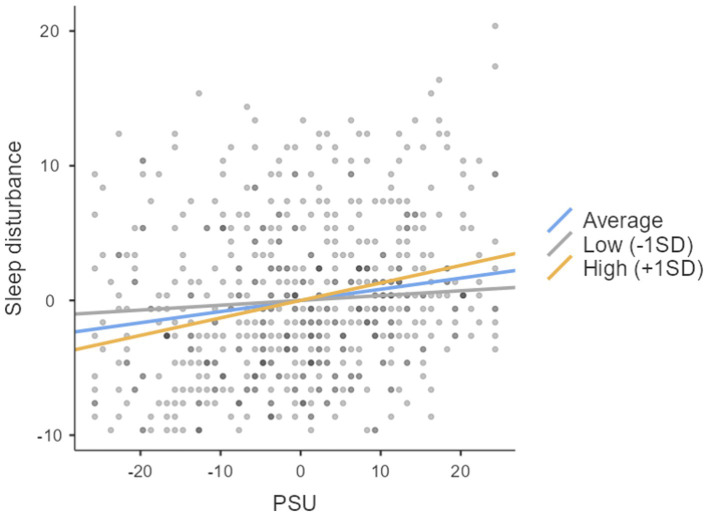
The moderating role of cognitive reappraisal at different levels.

Interestingly, cognitive reappraisal did not significantly predict sleep disturbance (see Table 4). However, its moderation role was significant. The association between PSU and sleep disturbance was found to be different at different levels of cognitive reappraisal; the slope shows that it was (0.15) at the low level, it was (0.05) at the high level, and it was (0.10) at the average. Figure 2 depicts the moderating role of cognitive reappraisal at different levels. As with expressive suppression, the strongest influence of cognitive reappraisal was at its low and average level.

#### 3.1.3. Verbal perseveration as a moderator

As seen in Table 4, verbal perseveration significantly and positively predicted sleep disturbance; however, it was not a significant moderator of the association between PSU and sleep disturbance. Consequently, the moderating role of verbal perseveration at different levels was not investigated.

#### 3.1.4. Imaginal prefiguration as a moderator

**Figure 3:**
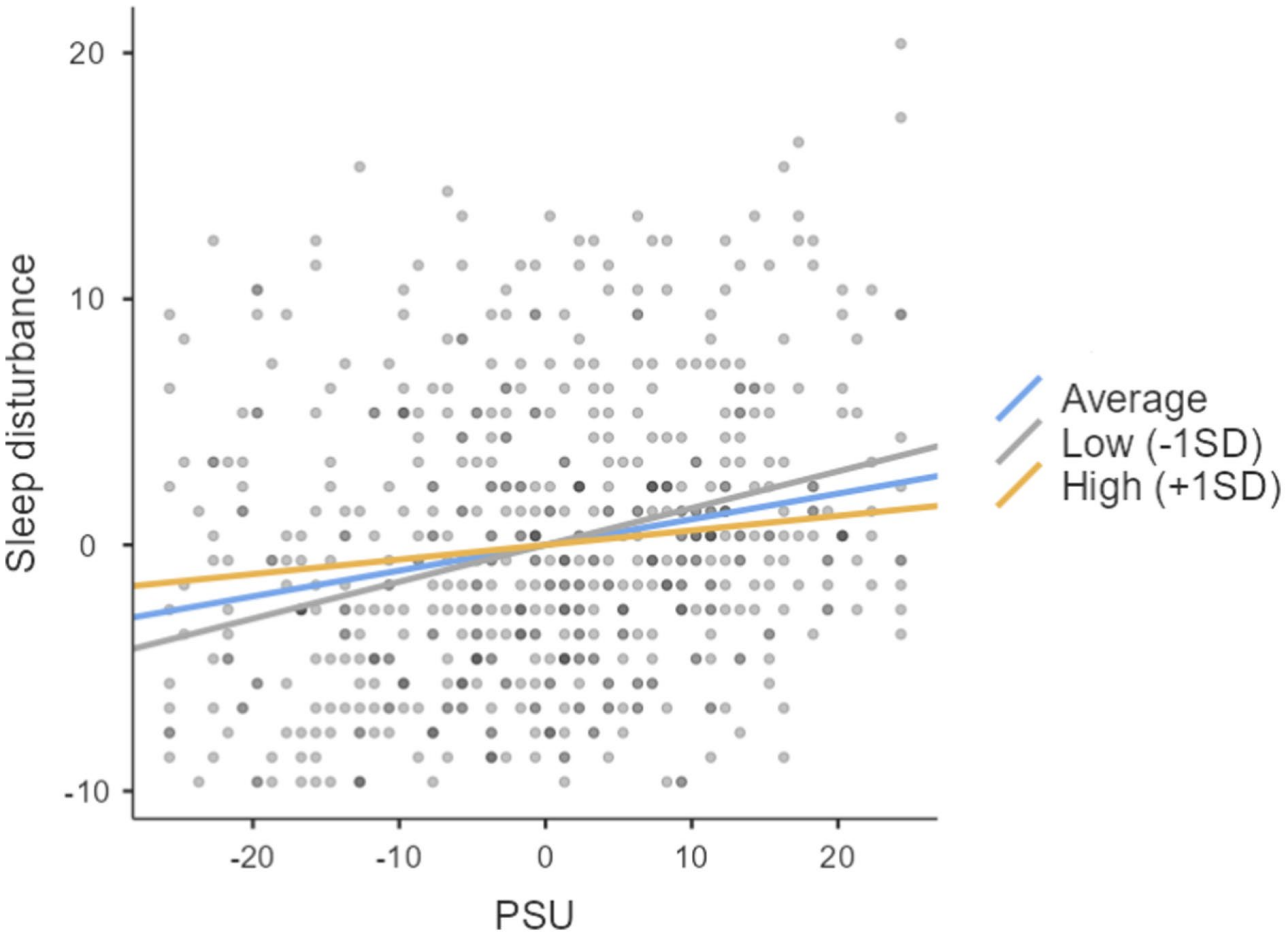
The moderating role of imaginal prefiguration at different levels.

Interestingly, imaginal prefiguration did not significantly predict sleep disturbance; however, it significantly moderated the association between PSU and sleep disturbance. The slope for the low level was not significant. However, it was significant at a high level (0.12) and average (0.08) level. The slope indicates that most influence of imaginal prefiguration is at its high level and then its average level. Figure 3 depicts the moderating role of imaginal prefiguration at different levels (see Table 5).

**Table 5:** Slope analysis based on a different level of moderators, with sleep disturbance as the dependent variable.

Variable	*B 95%CI* [Ul, Ll]	SE	*Z*	*p*
Model 1 – Suppression as a moderator				
Average	0.10 [0.06, 0.14]	0.0204	5.13	< 0.001
Low (-1SD)	0.15 [0.09, 0.20]	0.0302	4.95	< 0.001
High (+1SD)	0.06 [0.009, 0.10]	0.0261	2.27	0.023
Model 2 – Reappraisal as a moderator				
Average	0.10 [0.06, 0.14]	0.0205	5.12	< 0.001
Low (-1SD)	0.15 [0.10, 0.20]	0.0287	5.29	< 0.001
High (+1SD)	0.05 [0.007, 0.10]	0.0272	2.12	0.034
Model 3 – Imaginal Prefiguration as a moderator				
Average	0.08 [0.04, 0.11]	0.0262	3.16	0.002
Low (-1SD)	0.03 [−0.01, 0.08]	0.0276	1.30	0.194
High (+1SD)	0.12 [0.08, 0.17]	0.0407	3.19	0.001

## 4. Discussion

Given that PSU is a predictor of sleep disturbance and that metacognitions about smartphones, desire thinking, and emotion regulation are associated with PSU, the current study sought to investigate whether these variables interact with PSU in predicting sleep disturbance.

All factors of the MSUQ were positively and significantly associated with PSU and sleep disturbance. However, when the PSU level was accounted for, the MSUQ factors were no longer predictors of sleep disturbance which led to their omission in the interaction analysis. This finding is interesting given that in a pairwise association, it was observed that higher scores on the MSUQ factors were associated with higher scores on PSU and sleep disturbance, but additional variance in sleep disturbance could not be explained when PSU was controlled for. It might be argued that the non-significant role of the MSUQ factors is due to their non-linear effects on sleep disturbance, for example, they may be non-linear (e.g., curvilinear) moderators of the association between PSU and sleep disturbance which could not be examined using linear regression analysis [for more information on curvilinear moderators, please see (50)], and requires further investigation. Another potential explanation for this null effect might be explained by the notion of prediction interval -in some populations, an effect might be (a) null, (b) in the expected direction, or (c) even reverse (51), as some well-known associations might not be observed in all samples, for example, fear of missing out is not associated with Facebook use in some populations (52). That said, the MSUQ might explain additional variance in PSU in predicting sleep disturbance in other samples.

The findings show that desire thinking (verbal perseveration) predicted sleep disturbance above and beyond PSU, suggesting that self-talk regarding reasons for engaging in Smartphone use is a stronger predictor of sleep disturbance and it is above and beyond merely the excessive use of a Smartphone, i.e., the higher the desire (motivated by personal reasons) to use a Smartphone the higher the sleep disturbance. However, the effect of the imaginal prefiguration factor of desire thinking in predicting sleep disturbance was not significant, suggesting that constructing mental images on what Smartphones can afford is not a significant predictor of sleep disturbance when PSU, metacognitions about Smartphone use and verbal perseveration are controlled for. Interestingly, the moderation analysis revealed that verbal perseveration has no interaction with PSU in predicting sleep disturbance, while imaginal prefiguration when PSU was not controlled for, was found to be a significant predictor of sleep disturbance at the average score and high score (+1SD) and not low score (-1SD). These findings suggest that verbal perseveration uniquely contributes to sleep disturbance, while imaginal prefiguration only at the average and high scores could contribute to sleep disturbance exclusively by interaction with PSU.

Emotion regulation (expressive suppression and cognitive reappraisal) was also a significant predictor of sleep disturbance when it was added to the hierarchical regression analysis. This said, verbal perseveration remained the strongest predictor of sleep disturbance, but the effect of PSU become non-significant which might suggest the importance of emotion regulation in explaining sleep disturbance. However, when it comes to the moderator analysis when PSU was controlled for, expressive suppression was still a predictor of sleep disturbance, but this was not the case for cognitive reappraisal, suggesting that expressive suppression is a unique predictor of sleep disturbance. Nonetheless, the interactions between emotion regulation factors with PSU were significant, suggesting that cognitive reappraisal only by interacting with PSU could predict sleep disturbance, but expressive suppression by itself and by interacting with PSU could do the same. The slope analysis revealed that the association between PSU and sleep disturbance is at its highest, moderate, and lowest value when the suppression score is low (-1SD), average, and high (+1SD). This could be explained by the fact that expressive suppression is an emotional regulation strategy that has a sympathetic activation effect by increasing emotional arousal which substitutes appropriate emotion regulation (53). When it is at its lowest and average value, the association between PSU and sleep disturbance is substantial, i.e., PSU still maintains its effect, but when it is at the high level, the association between PSU and sleep disturbance would be negligible which might be a sign of the unique effect of expressive suppression on continuing sleep disturbance by increasing emotional arousal. This explanation is supported by the hierarchical regression analysis which shows that when adding emotion regulation factors in the model the effect of PSU in predicting sleep disturbance becomes non-significant.

Concerning cognitive reappraisal, although the slope analysis revealed the same result compared with expressive suppression, the association between PSU and sleep disturbance is at its highest, moderate, and lowest value when the cognitive reappraisal score is low (-1SD), average, and high (+1SD). This suggests that the association between PSU and sleep disturbance among people with low and average scores on cognitive reappraisal is higher, but it is negligible when the cognitive reappraisal score is high. Accordingly, expressive suppression and cognitive reappraisal both could reduce the association between PSU and sleep disturbance, however, theoretically, only cognitive reappraisal could protect against sleep disturbance in people with high scores on cognitive reappraisal. This said, it appears that expressive suppression, by decreasing the strength of association between PSU and sleep disturbance, by itself may maintain sleep disturbance given that the effect of PSU on sleep disturbance becomes non-significant when emotion regulation factors are added to the hierarchical regression model.

### 4.1. Limitations and future directions

All findings should be interpreted in light of the study’s limitations. Self-report measures can be influenced by recall bias and data gathering during the COVID-19 pandemic may have inflated the strengths of the estimation provided, given that Iranians experienced considerable psychological distress and pandemic related anxiety (54, 55). Moreover, the findings might be limited in generalization only to Iran and countries with similar demographics such as lower-middle income level countries and not to individuals from Western, Educated, Industrialized, Rich, and Democratic (WEIRD) nations (56). Also, given the cross-sectional nature of the findings, it is not possible to conclude a causal association between the variables, thus, bi-directionality between variables also needs to be considered. However, our work lays ground for further research. Researchers might want to replicate the moderating effects of metacognitions about Smartphone use in the association between PSU and sleep disturbance to see whether the observed null-effect might be better explained by prediction intervals as discussed previously. In addition, it would be useful if participants could be interviewed for sleep disorders and to see whether a different result could be found in confirmed cases of sleep disorder and not only sleep disturbance. Moreover, because we did not ask the participants about their state of emotion regulation, desire thinking, or metacognition about Smartphones, particularly when attempting to fall asleep, it is recommended that future studies use ecological momentary assessment to investigate the role of the aforementioned variables in this context and with greater specificity. Due to the cross-sectional nature of the findings, it is not feasible to infer a causal association between the variables; therefore, bidirectionality between variables must also be considered. Further, future studies may want to employ longitudinal designs to ensure the temporality of the observed moderation effects. Finally, given that cognitive reappraisal and expressive suppression both at high levels dampened the association between PSU and sleep disturbance it is worthy of more investigation to examine whether this finding could be replicated.

### 4.2. Conclusion

Overall, PSU was a predictor of sleep disturbance and while metacognitions about Smartphone use was linearly associated with sleep disturbance but they were not significant predictors of sleep disturbance when the PSU was controlled for. In addition, verbal perseveration and expressive suppression were unique predictors of sleep disturbance when the PSU was controlled, while imaginal prefiguration and cognitive reappraisal only could predict sleep disturbance if they interact with PSU. Given the significant interaction between desire thinking and emotion regulation with PSU in predicting sleep disturbance, it is recommended that clinicians assess for imaginal prefiguration and cognitive reappraisal because, theoretically, findings suggest that enhancing cognitive reappraisal (by 1 SD) and reducing imaginal prefiguration (by 1 SD), might protect against sleep disturbance by reducing its association with PSU.

## Data availability statement

The raw data supporting the conclusions of this article will be made available by the authors, without undue reservation.

## Ethics statement

The studies involving human participants were reviewed and approved by Kharazmi university. The patients/participants provided their written informed consent to participate in this study.

## Author contributions

MA, MoS, SS, and MaS: conception and drafting of the work. SS, MA, and MoS: data acquisition. MoS and MA: data analysis. MA, MoS, SS, and MaS: data interpretation. MA, MaS, and MoS: revising the manuscript critically for important intellectual content. All authors agreed to be accountable for all aspects of the work in ensuring that questions related to the accuracy or integrity of any part of the work are appropriately investigated and resolved and they approved the final version to be published.

## Conflict of interest

The authors declare that the research was conducted in the absence of any commercial or financial relationships that could be construed as a potential conflict of interest.

The reviewer GM declared a past collaboration with the authors to the handling Editor.

## Publisher’s note

All claims expressed in this article are solely those of the authors and do not necessarily represent those of their affiliated organizations, or those of the publisher, the editors and the reviewers. Any product that may be evaluated in this article, or claim that may be made by its manufacturer, is not guaranteed or endorsed by the publisher.
